# Subject: Motivation can be suppressed, but scientific ability cannot and should not be ignored

**DOI:** 10.1186/s12967-023-04383-1

**Published:** 2023-08-02

**Authors:** Najmaldin Saki, Habib Haybar, Mojtaba Aghaei

**Affiliations:** 1 Thalassemia & Hemoglobinopathy Research Center, Health Research Institute, Ahvaz Jundishapur University of Medical Sciences, Ahvaz, Iran; 2 Atherosclerosis Research Center, Ahvaz Jundishapur University of Medical Sciences, Ahvaz, Iran; 3 Student Research Committee, Ahvaz Jundishapur University of Medical Sciences, Ahvaz, Iran

Dear editor of “Journal of Translational Medicine”,

Medical researchers in different countries are an integral part of the world’s research community. According to the faculty bestowed to researchers by God and the task assigned to them, they work to better understand and help improve the level of health, treatment and medical education. Therefore, cultural, ethnic, racial and political differences and other current conflicts should not be a factor repelling and suppressing medical science research and even slowing down its course. Medical science plays a central role in society because it underpins advancing our understanding of disease, developing life-saving treatments, and improving health care [1]. COVID-19 pandemic was an important experience that taught us lessons about the significance of the health care systems. One of the most important lessons was that when medical resources and infrastructure in a particular region are inadequate or poorly managed, it can have devastating consequences for the entire global community [2]. We observe this consequence in many countries where inadequate supplies of personal protective equipment, limited testing capacity, and shortage of hospital beds and ventilators have resulted in high workloads for the health care system and a high rate of fatalities [3]. Ignoring the work of medical researchers can have dangerous consequences. Without their tireless efforts, we risk stagnation in medical knowledge, limitations in the exchange of experiences, restricted access to innovative treatments, and ultimately compromised patient care [4]. As emphasized by WHO, neglecting scientific research in medicine hinders progress, increases mortality rates, and impairs our ability to effectively respond to emerging health challenges [5]. Therefore, it is imperative that society supports the valuable contributions of all medical researchers to ensure the well-being of individuals and the advancement of health care in general.

The phenotype of thoughts in a society/individual is a function of the genotype of the books they have read (Dr. Saki); cultural differences can inevitably lead to misunderstandings and mistrust between researchers, which is an obstacle to progress and cooperation as well as sharing of ideas [6]. This is especially true in medicine and public health where the importance of cultural and religious variations in the diagnosis and treatment of disease cannot be ignored. Merely having a large number of scientists is not enough to develop a truly inclusive scientific community. We need to provide all of them with the resources and support systems they need to thrive. Nevertheless, these policies go beyond providing research funding and must include comprehensive training programs and valuable mentoring by experienced researchers [7]. It is not necessarily the case that a country with a large amount of oil reserves will allocate its revenue to research. Whereas possessing significant natural resources can provide a financial advantage, it does not guarantee the allocation of funds to research projects. The decision to allot resources to research is often influenced by political considerations and priorities. However, the political approach and attitude should not be attributed to researchers who are attempting to pursue their work with the abilities they have. It is of importance that scientific research remains independent of political influence because it is essential for the advancement of knowledge and progress. It is unfortunate that researchers in Ukraine and Russia are facing obstacles in conducting their research. The destruction and abandonment of some laboratories in Ukraine as well as their denial of research indicates the importance of maintaining proper infrastructure to facilitate scientific research, and in the same way, researchers in Russia have recently faced challenges due to increased costs and sanctions. Iranian students, professors and researchers have been struggling with these problems for several years in spite of the progress they have made. It is essential that governments and institutions recognize the vital role of research in advancing knowledge and solving real-world problems and allocate the necessary resources to support this important work.

Despite these daunting challenges for students and professors and acknowledging that motivation can be undermined, the potential and abilities of talented researchers from all cultures cannot be ignored. As a scientific community, we should not be prone to superficial comparisons or unjustifiable sanctions because such actions lead to more discord and we must step as a unified and harmonious body towards a better and brighter future for the next generation. Another point is that the idea of concentrating scientists and facilities in one region may seem good at first because it can foster collaboration and innovation. However, this is not a sustainable model for long-term scientific progress as it could lead to unequal distribution of human and physical resources. Decentralization can prevent the accumulation of resources in a region, country or laboratory and cause a fairer distribution of both knowledge and facilities.

Encouraging collaboration and exchange of ideas between researchers from different cultures is of high importance. This can be achieved through the availability of various methods such as international conferences, joint research projects, and exchange programs. Creating a network of young and veteran researchers is essential to prevent the risks arising from science. To reach this goal, we must have a multifaceted approach. First, we have to identify and contact potential candidates for the network and invite them to share their unique skills and perspectives. Second, we need to create a mentoring system where experienced researchers can guide and support young scientists in their work. Finally, a seat in important international bodies such as United Nations General Assembly should be established to raise awareness of the importance of scientific research and the risks associated with it. In this way, we can develop comprehensive cooperation for the advancement of science at the global level and eventually improve education, strengthen scientific cooperation and increase the level of health.

Email: najmaldinSaki@gmail.com

Phone No.: (+98) 9161143369

Thalassemia and Hemoglobinopathy Research Center, Health research institute, Ahvaz Jundishapur University of Medical Sciences, Ahvaz, Iran.

## Data Availability

Not applicable.
